# Triaqua­(2,2′-bipyridine *N*,*N*′-dioxide-κ^2^ *O*,*O*′)(5-nitro­benzene-1,3-dicarboxyl­ato-κ*O* ^1^)zinc(II)

**DOI:** 10.1107/S1600536809015451

**Published:** 2009-05-20

**Authors:** Hui-Juan Lu, Fang-Ming Wang

**Affiliations:** a Department of Chemical Engineering, Wuhan University of Science and Engineering, Wuhan, Hubei 430073, People’s Republic of China; b School of Materials Science and Engineering, Jiangsu University of Science and Technology, Zhenjiang, Jiangsu 212003, People’s Republic of China

## Abstract

In the title compound, [Zn(C_8_H_3_NO_6_)(C_10_H_8_N_2_O_2_)(H_2_O)_3_], the Zn^II^ ion is coordinated in a distorted octa­hedral geometry by three water mol­ecules, one O atom from a 5-nitro­benzene-1,3-dicarboxyl­ate ligand and two O atoms from a chelating 2,2′-bipyridine *N*,*N*′-dioxide ligand. An extensive network of O—H⋯O hydrogen bonds is formed between the water mol­ecules and the carboxyl­ate groups. C—H⋯O inter­actions are also present.

## Related literature

For metal complexes containing the 2,2′-bipyridine-*N*,*N*′-dioxide ligand, see: Hill *et al.* (2004 ▶); Long *et al.* (2001 ▶); Ma *et al.* (2003 ▶).
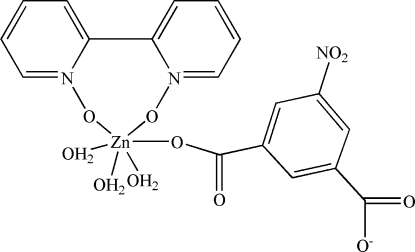


## Experimental

### 

#### Crystal data

[Zn(C_8_H_3_NO_6_)(C_10_H_8_N_2_O_2_)(H_2_O)_3_]
*M* *_r_* = 516.72
Triclinic, 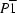

*a* = 8.3040 (14) Å
*b* = 10.7036 (18) Å
*c* = 11.6546 (19) Å
α = 87.217 (3)°
β = 88.436 (3)°
γ = 87.006 (3)°
*V* = 1032.9 (3) Å^3^
*Z* = 2
Mo *K*α radiation
μ = 1.26 mm^−1^
*T* = 294 K
0.20 × 0.19 × 0.15 mm

#### Data collection

Bruker SMART CCD area-detector diffractometer
Absorption correction: multi-scan (*SADABS*; Sheldrick, 1996 ▶) *T*_min_ = 0.778, *T*_max_ = 0.828
6281 measured reflections
3604 independent reflections
2737 reflections with *I* > 2σ(*I*)
*R*_int_ = 0.075

#### Refinement

*R*[*F* ^2^ > 2σ(*F* ^2^)] = 0.054
*wR*(*F* ^2^) = 0.143
*S* = 1.05
3604 reflections
316 parameters
15 restraints
H atoms treated by a mixture of independent and constrained refinement
Δρ_max_ = 0.62 e Å^−3^
Δρ_min_ = −0.52 e Å^−3^

### 

Data collection: *SMART* (Bruker, 2001 ▶); cell refinement: *SAINT-Plus* (Bruker, 2001 ▶); data reduction: *SAINT-Plus*; program(s) used to solve structure: *SHELXS97* (Sheldrick, 2008 ▶); program(s) used to refine structure: *SHELXL97* (Sheldrick, 2008 ▶); molecular graphics: *SHELXTL* (Sheldrick, 2008 ▶); software used to prepare material for publication: *SHELXTL*.

## Supplementary Material

Crystal structure: contains datablocks I, global. DOI: 10.1107/S1600536809015451/gk2207sup1.cif

Structure factors: contains datablocks I. DOI: 10.1107/S1600536809015451/gk2207Isup2.hkl

Additional supplementary materials:  crystallographic information; 3D view; checkCIF report

## Figures and Tables

**Table 1:** Selected bond lengths (Å)

O1—Zn1	2.094 (3)
O2—Zn1	2.144 (3)
O3—Zn1	2.043 (3)
O9—Zn1	2.125 (3)
O10—Zn1	2.067 (3)
O11—Zn1	2.054 (4)

**Table 2:** Hydrogen-bond geometry (Å, °)

*D*—H⋯*A*	*D*—H	H⋯*A*	*D*⋯*A*	*D*—H⋯*A*
O9—H9*A*⋯O5^i^	0.82 (3)	2.08 (4)	2.773 (5)	143 (5)
O9—H9*B*⋯O4	0.83 (3)	1.83 (3)	2.635 (5)	164 (4)
O10—H10*A*⋯O6^ii^	0.81 (5)	1.96 (5)	2.735 (5)	161 (6)
O10—H10*B*⋯O6^iii^	0.82 (5)	2.03 (5)	2.702 (5)	139 (5)
O11—H11*A*⋯O2^iv^	0.82 (3)	1.95 (3)	2.687 (5)	149 (5)
O11—H11*B*⋯O5^ii^	0.83 (3)	1.87 (4)	2.692 (5)	169 (4)
C2—H2⋯O8^v^	0.93	2.59	3.229 (8)	126
C3—H3⋯O6^vi^	0.93	2.49	3.269 (7)	141
C4—H4⋯O3^vii^	0.93	2.46	3.358 (7)	162

Symmetry codes: (i) 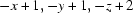; (ii) 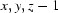; (iii) 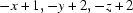; (iv) 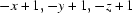; (v) 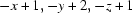; (vi) 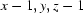; (vii) 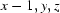.

**Fig. 1.**
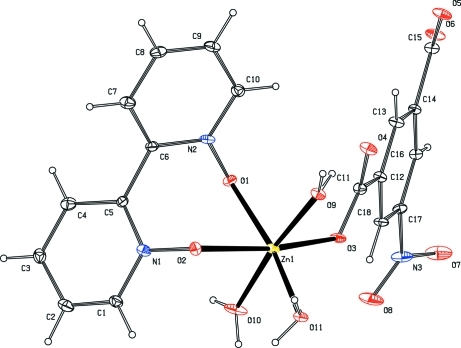
The molecular structure of the title compound, showing displacement ellipsoids at the 50% probability level.

**Fig. 2.**
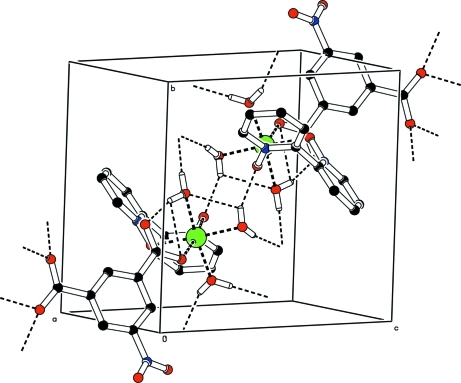
Part of the crystal structure showing hydrogen bonds. H atoms not involved in hydrogen bonding have been omitted.

## supplementary crystallographic information

### Comment

Herein we report the crystal structure of a novel compound, [Zn(C_10_H_8_N_2_O_2_)(C_8_H_3_NO_6_)(H_2_O)_3_](I). As shown in Fig.1, the central Zn atom is coordinated by three O atoms from three water molecules, one O atom from 5-nitrobenzene-1,3-dicarboxylate ligand and two O atoms from 2,2'-bipyridine-N,*N*'-dioxide ligand in a distorted octahedral geometry. The 2,2'-bipyridine-N,*N*'-dioxide coordinated to the Zn atom forms a seven-membered chalate ring. O1, O2, O3 and O11 atoms lie in the equatorial plane, with the O3—O1—O2—O11 torsional angle of 0.51°, while the Zn atom deviates from the equatorial plane by 0.026 Å. O9 and O10 atoms occupy the axial sites, with O9—Zn1—O10 angle of 173.04°) (distances to the equatorial plane are 2.143 and 2.040 Å). Among the distances of Zn—O, the distance of Zn(1)—O(2) is the longest, (see Table 2). The neighboring molecules in the crystal are linked by a series of O—H···O and C—H ···O intermolecular hydrogen bonds.

### Experimental

A mixture of ZnSO_4_(0.50 mmol),5-nitrobenzene-1,3-dicarboxylic acid (0.50 mmol), 2,2'-bipyridine-N,*N*'-dioxide (0.50 mmol), and H_2_O (3.00 ml), was placed in a Parr Teflon-lined stainless steel vessel (10 mL), and then the vessel was sealed and heated at 393 K for 3 days. After the mixture was slowly cooled to room temperature, several colourless crystals of the title compound were obtained.

### Refinement

H atoms of the water molecules were located in a difference Fourier map and refined with O—H distance restraints of 0.80 (2) Å,H···H distance restraints of 1.35 (4) Å and *U*_iso_(H) = 1.5*U*_eq_(O). H atoms bonded to C atoms were introduced at calculated positions and refined using a riding model, with *U*_iso_(H) = 1.2*U*_eq_(C) and C–H distancess of 0.93 Å. The displacement parameters of N1 and O2 were restrained with the SIMU function of SHELXL-97.

### Crystal data

**Table:**

[Zn(C_8_H_3_NO_6_)(C_10_H_8_N_2_O_2_)(H_2_O)_3_]	*Z* = 2
*M**_r_* = 516.72	*F*(000) = 528
Triclinic, *P*1	*D*_x_ = 1.661 Mg m^−^^3^
Hall symbol: -P 1	Mo *K*α radiation, λ = 0.71073 Å
*a* = 8.3040 (14) Å	Cell parameters from 1831 reflections
*b* = 10.7036 (18) Å	θ = 2.5–25.8°
*c* = 11.6546 (19) Å	µ = 1.26 mm^−^^1^
α = 87.217 (3)°	*T* = 294 K
β = 88.436 (3)°	Plate, colourless
γ = 87.006 (3)°	0.20 × 0.19 × 0.15 mm
*V* = 1032.9 (3) Å^3^	

### Data collection

**Table:**

Bruker SMART CCD area-detector diffractometer	3604 independent reflections
Radiation source: fine-focus sealed tube	2737 reflections with *I* > 2σ(*I*)
graphite	*R*_int_ = 0.075
Detector resolution: 0 pixels mm^-1^	θ_max_ = 25.0°, θ_min_ = 1.8°
φ and ω scans	*h* = −9→9
Absorption correction: multi-scan (*SADABS*; Sheldrick, 1996)	*k* = −12→12
*T*_min_ = 0.778, *T*_max_ = 0.828	*l* = −13→12
6281 measured reflections	

### Refinement

**Table:**

Refinement on *F*^2^	Primary atom site location: structure-invariant direct methods
Least-squares matrix: full	Secondary atom site location: difference Fourier map
*R*[*F*^2^ > 2σ(*F*^2^)] = 0.054	Hydrogen site location: inferred from neighbouring sites
*wR*(*F*^2^) = 0.143	H atoms treated by a mixture of independent and constrained refinement
*S* = 1.05	*w* = 1/[σ^2^(*F*_o_^2^) + (0.0678*P*)^2^] where *P* = (*F*_o_^2^ + 2*F*_c_^2^)/3
3604 reflections	(Δ/σ)_max_ = 0.001
316 parameters	Δρ_max_ = 0.62 e Å^−^^3^
15 restraints	Δρ_min_ = −0.52 e Å^−^^3^

### Special details

**Table:**

Geometry. All e.s.d.'s (except the e.s.d. in the dihedral angle between two l.s. planes) are estimated using the full covariance matrix. The cell e.s.d.'s are taken into account individually in the estimation of e.s.d.'s in distances, angles and torsion angles; correlations between e.s.d.'s in cell parameters are only used when they are defined by crystal symmetry. An approximate (isotropic) treatment of cell e.s.d.'s is used for estimating e.s.d.'s involving l.s. planes.
Refinement. Refinement of *F*^2^ against ALL reflections. The weighted *R*-factor *wR* and goodness of fit *S* are based on *F*^2^, conventional *R*-factors *R* are based on *F*, with *F* set to zero for negative *F*^2^. The threshold expression of *F*^2^ > σ(*F*^2^) is used only for calculating *R*-factors(gt) *etc*. and is not relevant to the choice of reflections for refinement. *R*-factors based on *F*^2^ are statistically about twice as large as those based on *F*, and *R*- factors based on ALL data will be even larger.

### Fractional atomic coordinates and isotropic or equivalent isotropic displacement parameters (Å^2^)

**Table:**

	*x*	*y*	*z*	*U*_iso_*/*U*_eq_	
C1	0.1974 (7)	0.7637 (5)	0.4162 (4)	0.0393 (13)	
H1	0.2819	0.7530	0.3629	0.047*	
C2	0.0764 (7)	0.8456 (5)	0.3923 (5)	0.0501 (15)	
H2	0.0790	0.8941	0.3239	0.060*	
C3	−0.0552 (7)	0.8599 (5)	0.4689 (5)	0.0422 (14)	
H3	−0.1426	0.9146	0.4510	0.051*	
C4	−0.0514 (7)	0.7907 (5)	0.5715 (5)	0.0419 (13)	
H4	−0.1365	0.7988	0.6245	0.050*	
C5	0.0808 (6)	0.7082 (4)	0.5958 (4)	0.0291 (11)	
C6	0.0829 (6)	0.6224 (4)	0.6979 (4)	0.0316 (11)	
C7	−0.0296 (7)	0.5359 (5)	0.7202 (5)	0.0446 (14)	
H7	−0.1152	0.5322	0.6710	0.054*	
C8	−0.0185 (7)	0.4531 (5)	0.8157 (5)	0.0467 (15)	
H8	−0.0946	0.3931	0.8298	0.056*	
C9	0.1038 (7)	0.4616 (5)	0.8867 (5)	0.0437 (14)	
H9	0.1116	0.4074	0.9514	0.052*	
C10	0.2200 (6)	0.5500 (5)	0.8659 (4)	0.0383 (12)	
H10	0.3051	0.5543	0.9155	0.046*	
C11	0.6375 (6)	0.7121 (4)	0.8717 (4)	0.0319 (11)	
C12	0.6641 (6)	0.8090 (4)	0.9588 (4)	0.0294 (11)	
C13	0.6310 (6)	0.7849 (4)	1.0732 (4)	0.0315 (12)	
H13	0.5928	0.7074	1.0966	0.038*	
C14	0.6522 (6)	0.8719 (4)	1.1562 (4)	0.0279 (11)	
C15	0.6128 (6)	0.8387 (4)	1.2805 (4)	0.0313 (12)	
C16	0.7084 (6)	0.9882 (4)	1.1206 (4)	0.0308 (11)	
H16	0.7229	1.0490	1.1732	0.037*	
C17	0.7419 (6)	1.0105 (4)	1.0055 (4)	0.0305 (11)	
C18	0.7202 (6)	0.9267 (5)	0.9228 (4)	0.0345 (12)	
H18	0.7417	0.9469	0.8456	0.041*	
N1	0.1998 (5)	0.6958 (4)	0.5163 (3)	0.0334 (10)	
N2	0.2069 (5)	0.6291 (4)	0.7726 (3)	0.0337 (10)	
N3	0.8039 (6)	1.1326 (4)	0.9671 (4)	0.0534 (14)	
O1	0.3113 (4)	0.7178 (3)	0.7539 (3)	0.0333 (8)	
O2	0.3271 (4)	0.6146 (3)	0.5377 (3)	0.0321 (8)	
O3	0.6489 (4)	0.7466 (3)	0.7662 (3)	0.0352 (8)	
O4	0.6108 (5)	0.6041 (3)	0.9116 (3)	0.0420 (9)	
O5	0.5983 (5)	0.7257 (3)	1.3088 (3)	0.0444 (10)	
O6	0.5973 (5)	0.9246 (3)	1.3487 (3)	0.0473 (10)	
O7	0.8838 (7)	1.1868 (4)	1.0367 (4)	0.0822 (16)	
O8	0.7846 (7)	1.1702 (4)	0.8683 (4)	0.0804 (16)	
O9	0.5579 (5)	0.4929 (3)	0.7204 (3)	0.0339 (8)	
H9A	0.546 (7)	0.418 (2)	0.732 (4)	0.051*	
H9B	0.562 (7)	0.518 (4)	0.786 (2)	0.051*	
O10	0.4917 (6)	0.8518 (3)	0.5648 (3)	0.0561 (12)	
H10A	0.544 (7)	0.872 (6)	0.509 (4)	0.084*	
H10B	0.421 (6)	0.904 (5)	0.583 (5)	0.084*	
O11	0.6736 (4)	0.6260 (3)	0.5182 (3)	0.0326 (8)	
H11A	0.640 (6)	0.556 (2)	0.514 (4)	0.049*	
H11B	0.644 (6)	0.664 (4)	0.458 (3)	0.049*	
Zn1	0.50994 (7)	0.67555 (5)	0.64551 (4)	0.0280 (2)	

### Atomic displacement parameters (Å^2^)

**Table:**

	*U*^11^	*U*^22^	*U*^33^	*U*^12^	*U*^13^	*U*^23^
C1	0.045 (3)	0.047 (3)	0.025 (3)	0.002 (3)	0.001 (2)	0.001 (2)
C2	0.064 (4)	0.049 (4)	0.036 (3)	−0.006 (3)	−0.001 (3)	0.014 (3)
C3	0.039 (3)	0.034 (3)	0.053 (4)	−0.002 (2)	−0.009 (3)	0.005 (3)
C4	0.049 (3)	0.033 (3)	0.045 (3)	−0.016 (3)	0.003 (3)	−0.002 (3)
C5	0.025 (3)	0.031 (3)	0.032 (3)	−0.003 (2)	0.002 (2)	0.000 (2)
C6	0.032 (3)	0.031 (3)	0.031 (3)	−0.005 (2)	0.005 (2)	0.005 (2)
C7	0.055 (4)	0.039 (3)	0.041 (3)	−0.012 (3)	0.003 (3)	0.004 (3)
C8	0.051 (4)	0.037 (3)	0.052 (4)	−0.015 (3)	0.011 (3)	0.007 (3)
C9	0.059 (4)	0.035 (3)	0.035 (3)	−0.002 (3)	0.009 (3)	0.011 (2)
C10	0.041 (3)	0.042 (3)	0.032 (3)	0.003 (2)	0.003 (2)	−0.002 (2)
C11	0.039 (3)	0.032 (3)	0.026 (3)	−0.004 (2)	0.001 (2)	−0.007 (2)
C12	0.040 (3)	0.025 (3)	0.022 (3)	−0.003 (2)	0.001 (2)	0.000 (2)
C13	0.053 (3)	0.017 (2)	0.024 (3)	−0.004 (2)	0.005 (2)	−0.004 (2)
C14	0.036 (3)	0.027 (3)	0.020 (2)	−0.002 (2)	0.003 (2)	0.000 (2)
C15	0.046 (3)	0.021 (3)	0.027 (3)	−0.006 (2)	0.005 (2)	−0.007 (2)
C16	0.036 (3)	0.027 (3)	0.029 (3)	−0.005 (2)	0.004 (2)	−0.004 (2)
C17	0.037 (3)	0.021 (2)	0.034 (3)	−0.010 (2)	0.009 (2)	−0.002 (2)
C18	0.045 (3)	0.038 (3)	0.021 (3)	−0.011 (2)	0.008 (2)	0.005 (2)
N1	0.049 (3)	0.028 (2)	0.024 (2)	−0.0079 (19)	−0.001 (2)	−0.0047 (18)
N2	0.048 (3)	0.028 (2)	0.025 (2)	−0.0071 (19)	0.012 (2)	0.0035 (18)
N3	0.083 (4)	0.034 (3)	0.043 (3)	−0.021 (3)	0.028 (3)	−0.007 (2)
O1	0.0317 (18)	0.035 (2)	0.0343 (19)	−0.0105 (15)	0.0064 (15)	−0.0039 (15)
O2	0.0346 (19)	0.0302 (19)	0.0315 (19)	0.0060 (15)	−0.0029 (15)	−0.0078 (15)
O3	0.056 (2)	0.035 (2)	0.0162 (17)	−0.0189 (17)	0.0065 (16)	−0.0040 (14)
O4	0.075 (3)	0.027 (2)	0.0250 (19)	−0.0127 (18)	−0.0032 (18)	−0.0032 (15)
O5	0.089 (3)	0.0219 (19)	0.0234 (19)	−0.0153 (18)	0.0033 (19)	0.0003 (14)
O6	0.089 (3)	0.028 (2)	0.0233 (19)	−0.0018 (19)	0.0155 (19)	−0.0030 (16)
O7	0.141 (5)	0.055 (3)	0.056 (3)	−0.055 (3)	0.033 (3)	−0.023 (2)
O8	0.136 (5)	0.056 (3)	0.051 (3)	−0.041 (3)	0.012 (3)	0.019 (2)
O9	0.054 (2)	0.0217 (18)	0.0266 (19)	−0.0063 (17)	−0.0021 (17)	−0.0016 (15)
O10	0.118 (4)	0.021 (2)	0.028 (2)	−0.003 (2)	0.026 (2)	−0.0001 (16)
O11	0.048 (2)	0.0273 (19)	0.0229 (19)	−0.0100 (17)	0.0039 (16)	0.0005 (15)
Zn1	0.0418 (4)	0.0228 (3)	0.0197 (3)	−0.0056 (2)	0.0028 (2)	−0.0024 (2)

### Geometric parameters (Å, °)

**Table:**

C1—C2	1.325 (7)	C13—H13	0.9300
C1—N1	1.344 (6)	C14—C16	1.393 (6)
C1—H1	0.9300	C14—C15	1.507 (6)
C2—C3	1.399 (8)	C15—O6	1.243 (5)
C2—H2	0.9300	C15—O5	1.249 (5)
C3—C4	1.375 (7)	C16—C17	1.374 (6)
C3—H3	0.9300	C16—H16	0.9300
C4—C5	1.398 (7)	C17—C18	1.370 (6)
C4—H4	0.9300	C17—N3	1.475 (6)
C5—N1	1.342 (6)	C18—H18	0.9300
C5—C6	1.468 (6)	N1—O2	1.353 (5)
C6—C7	1.361 (7)	N2—O1	1.325 (5)
C6—N2	1.374 (6)	N3—O8	1.214 (6)
C7—C8	1.390 (7)	N3—O7	1.244 (6)
C7—H7	0.9300	O1—Zn1	2.094 (3)
C8—C9	1.338 (7)	O2—Zn1	2.144 (3)
C8—H8	0.9300	O3—Zn1	2.043 (3)
C9—C10	1.394 (7)	O9—Zn1	2.125 (3)
C9—H9	0.9300	O9—H9A	0.816 (19)
C10—N2	1.349 (6)	O9—H9B	0.821 (19)
C10—H10	0.9300	O10—Zn1	2.067 (3)
C11—O4	1.254 (5)	O10—H10A	0.80 (2)
C11—O3	1.268 (5)	O10—H10B	0.818 (19)
C11—C12	1.514 (6)	O11—Zn1	2.054 (4)
C12—C13	1.370 (6)	O11—H11A	0.814 (19)
C12—C18	1.407 (6)	O11—H11B	0.833 (19)
C13—C14	1.395 (6)		
			
C2—C1—N1	120.9 (5)	C17—C16—C14	118.1 (4)
C2—C1—H1	119.5	C17—C16—H16	121.0
N1—C1—H1	119.5	C14—C16—H16	121.0
C1—C2—C3	120.7 (5)	C18—C17—C16	124.3 (4)
C1—C2—H2	119.7	C18—C17—N3	117.2 (4)
C3—C2—H2	119.7	C16—C17—N3	118.6 (4)
C4—C3—C2	118.0 (5)	C17—C18—C12	117.7 (4)
C4—C3—H3	121.0	C17—C18—H18	121.2
C2—C3—H3	121.0	C12—C18—H18	121.2
C3—C4—C5	119.9 (5)	C5—N1—C1	121.6 (5)
C3—C4—H4	120.1	C5—N1—O2	119.5 (4)
C5—C4—H4	120.1	C1—N1—O2	118.9 (4)
N1—C5—C4	118.8 (5)	O1—N2—C10	120.1 (4)
N1—C5—C6	118.7 (4)	O1—N2—C6	118.9 (4)
C4—C5—C6	122.0 (4)	C10—N2—C6	121.0 (4)
C7—C6—N2	119.1 (5)	O8—N3—O7	123.8 (5)
C7—C6—C5	123.2 (5)	O8—N3—C17	118.8 (5)
N2—C6—C5	117.8 (4)	O7—N3—C17	117.2 (5)
C6—C7—C8	121.0 (5)	N2—O1—Zn1	116.6 (3)
C6—C7—H7	119.5	N1—O2—Zn1	117.1 (2)
C8—C7—H7	119.5	C11—O3—Zn1	121.9 (3)
C9—C8—C7	118.6 (5)	Zn1—O9—H9A	157 (4)
C9—C8—H8	120.7	Zn1—O9—H9B	93 (3)
C7—C8—H8	120.7	H9A—O9—H9B	102 (4)
C8—C9—C10	121.4 (5)	Zn1—O10—H10A	124 (4)
C8—C9—H9	119.3	Zn1—O10—H10B	122 (4)
C10—C9—H9	119.3	H10A—O10—H10B	114 (5)
N2—C10—C9	119.0 (5)	Zn1—O11—H11A	94 (4)
N2—C10—H10	120.5	Zn1—O11—H11B	107 (4)
C9—C10—H10	120.5	H11A—O11—H11B	104 (4)
O4—C11—O3	126.4 (4)	O3—Zn1—O11	103.59 (13)
O4—C11—C12	116.2 (4)	O3—Zn1—O10	88.62 (16)
O3—C11—C12	117.4 (4)	O11—Zn1—O10	87.49 (15)
C13—C12—C18	118.8 (4)	O3—Zn1—O1	86.88 (13)
C13—C12—C11	120.9 (4)	O11—Zn1—O1	169.46 (12)
C18—C12—C11	120.3 (4)	O10—Zn1—O1	91.57 (15)
C12—C13—C14	122.7 (4)	O3—Zn1—O9	89.47 (13)
C12—C13—H13	118.6	O11—Zn1—O9	86.47 (13)
C14—C13—H13	118.6	O10—Zn1—O9	173.05 (17)
C16—C14—C13	118.4 (4)	O1—Zn1—O9	95.00 (13)
C16—C14—C15	121.9 (4)	O3—Zn1—O2	169.33 (13)
C13—C14—C15	119.7 (4)	O11—Zn1—O2	86.92 (12)
O6—C15—O5	124.0 (5)	O10—Zn1—O2	90.04 (16)
O6—C15—C14	118.4 (4)	O1—Zn1—O2	82.58 (12)
O5—C15—C14	117.6 (4)	O9—Zn1—O2	93.05 (13)

### Hydrogen-bond geometry (Å, °)

**Table:**

*D*—H···*A*	*D*—H	H···*A*	*D*···*A*	*D*—H···*A*
O9—H9A···O5^i^	0.82 (3)	2.08 (4)	2.773 (5)	143 (5)
O9—H9B···O4	0.83 (3)	1.83 (3)	2.635 (5)	164 (4)
O10—H10A···O6^ii^	0.81 (5)	1.96 (5)	2.735 (5)	161 (6)
O10—H10B···O6^iii^	0.82 (5)	2.03 (5)	2.702 (5)	139 (5)
O11—H11A···O2^iv^	0.82 (3)	1.95 (3)	2.687 (5)	149 (5)
O11—H11B···O5^ii^	0.83 (3)	1.87 (4)	2.692 (5)	169 (4)
C2—H2···O8^v^	0.93	2.59	3.229 (8)	126
C3—H3···O6^vi^	0.93	2.49	3.269 (7)	141
C4—H4···O3^vii^	0.93	2.46	3.358 (7)	162
C13—H13···O4i	0.93	2.47	2.780 (6)	100

Symmetry codes: (i) −*x*+1, −*y*+1, −*z*+2; (ii) *x*, *y*, *z*−1; (iii) −*x*+1, −*y*+2, −*z*+2; (iv) −*x*+1, −*y*+1, −*z*+1; (v) −*x*+1, −*y*+2, −*z*+1; (vi) *x*−1, *y*, *z*−1; (vii) *x*−1, *y*, *z*; i.
